# A Case Report on Refractory Moschcowitz Syndrome

**DOI:** 10.7759/cureus.2664

**Published:** 2018-05-21

**Authors:** Beenish Fayyaz, Hafiz Jawad Rehman

**Affiliations:** 1 Internal Medicine, GBMC

**Keywords:** moschcowitz syndrome, thrombotic thrombocytopenic purpura, thrombotic microangiopathy, multiorgan failure, plasmapheresis, rituximab

## Abstract

Although completely identified later on, Thrombotic thrombocytopenic purpura was first described by Dr. Moschcowitz in 1924, therefore the name 'Moschcowitz syndrome'. It is a microangiopathy associated with thrombocytopenia and hemolysis which causes organ dysfunction secondary to widespread microvascular thrombosis. The spectrum of the organ dysfunction in thrombotic thrombocytopenic purpura is quite diverse and not limited to nervous system and kidneys as classically described thus making the diagnosis more difficult. Use of plasmapheresis has led to improvement in mortality rates associated with this disease. However, multiple organ failure and presence of shock indicates likelihood of severe disease activity or refractoriness which can be avoided with earlier initiation of immunosuppressive therapy such as Rituximab.

## Introduction

Moschcowitz syndrome, more commonly known as Thrombotic thrombocytopenic purpura (TTP), is a primary thrombotic micro-angiopathic disorder which is characterized by the classical 'pentad' of fever, thrombocytopenia, micro-angiopathic hemolytic anemia (MAHA), renal failure and neurological dysfunction [1]. However, a review of the literature indicates that this 'pentad' is not that commonly associated with TTP. While physicians and researchers continue to explore the pathophysiology of TTP, it has become evident that this disease can have various 'non-classical' manifestations leading to multiple organ failure. Although the advent of plasmapheresis has led to significant improvement in mortality rates, severe and refractory forms of TTP need to be managed more aggressively. Here, we report an adult female who presented with atypical features of TTP with a subsequent smoldering course which was managed with other modalities in addition to plasmapheresis and corticosteroids.

## Case presentation

**Figure 1:**
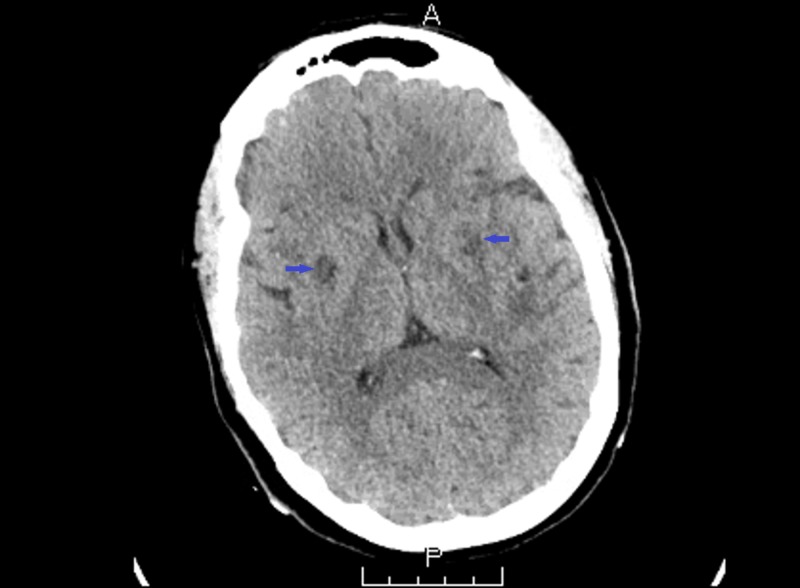
Computed tomography (CT) head non-contrast showing areas of diminished density in basal ganglia bilaterally (highlighted by blue arrows).

A 44-year-old female with no significant past medical history presented in the emergency department with complaints of fatigue, hematuria and nausea for two days. She had also noticed bruises present on her arms. On examination, she was hemodynamically stable. Blood workup showed hemoglobin of 9.9 grams per deciliter (g/dl), platelet count of 8000 per cubic millimeter (mm^3^), creatinine of 2.4 milligram per deciliter (mg/dl) and normal coagulation profile. Total bilirubin was 2.9 milligram per deciliter (mg/dl) with indirect bilirubin of 2.3 milligram per deciliter (mg/dl), Lactate dehydrogenase (LDH) of 1131 units per liter (IU/L) and haptoglobin of less than 10 milligram per deciliter (mg/dl). Urinalysis revealed proteinuria and hematuria along with granular casts. Due to complaints of chest discomfort, troponin levels were done which were found to be elevated at 1.43 nanogram per milliliter (ng/ml) although electrocardiogram (ECG) did not show any abnormalities. Meanwhile, peripheral smear demonstrated red cell fragmentation and schistocytes. As the laboratory values were indicative of a microangiopathic disorder, the patient was transferred to intensive care unit while ADAMTS-13 (A Disintegrin And Metalloprotease with ThromboSpondin type 1 motif-member 13) activity assays were requested. A central line was inserted and plasmapheresis was initiated along with intravenous corticosteroids. However, patient's condition deteriorated the next day and intubation was performed due to depressed mental level and hypotension. Blood workup showed platelet count of 2000 per cubic millimeter (mm^3^), Aspartate aminotransferase 1672 units per liter (IU/L), Alanine aminotransferase 1163 units per liter (IU/L), Lactic acid of 22.8 millimole per liter (mmol/L) and creatinine of 4.1 milligram per deciliter (mg/dl) with decline in urine output. Computed tomography (CT) head showed acute bilateral infarcts involving the basal ganglia (Figure 1).

At this time, the results of ADAMTS-13 assay depicted activity level of <5% with inhibitor levels >60% thus confirming acquired TTP. Hematology team decided to initiate Rituximab therapy in addition to plasmapheresis and corticosteroids due to initial inadequate response. Meanwhile, nephrology was consulted who advised to start CRRT (Continuous renal replacement therapy) for the progressive renal insufficiency. In view of rising troponin levels 1.43 to 3.6 nanogram per milliliter (ng/ml) and reduced ejection fraction of 45% plus global hypokinesia as seen on echocardiogram, she was suspected to have cardiogenic shock which was treated with vasopressors.

After being aggressively treated, the patient started showing clinical improvement by day five as evident by increasing platelet count of 48000 per cubic millimeter (mm^3^) and minimal vasopressor requirement. She was taken off vasopressors on day seven of admission and successfully extubated. As she continued to be hemodynamically stable, she was shifted to hemodialysis which was later on stopped due to adequate urine output and stable renal parameters. During 25 days of hospital stay, she received total four doses of Rituximab and 15 cycles of plasmapheresis before being discharged to a sub-acute rehab facility while on a steroid taper. Laboratory values at the time of discharge showed platelet count of 112,000 per cubic millimeter (mm^3^), creatinine of 1.7 milligram per deciliter (mg/dl) and ADAMTS-13 activity of 116%.

## Discussion

In 1924, Dr. Moschcowitz described the autopsy findings in a 16-year-old girl who died of petechiae and pallor followed by coma. He discovered hyaline thrombi in terminal arterioles and capillaries which he suspected were caused by a 'poison with agglutinative and hemolytic properties' [2]. That disease was later on identified as TTP which is clinically characterized by fever, thrombocytopenia, micro-angiopathic hemolytic anemia, renal insufficiency and neurological manifestations [1].

The pathophysiology of TTP revolves around an enzyme called ADAMTS-13 (A Disintegrin And Metalloprotease with ThromboSpondin type 1 motif-member 13) [3]. Normally, this protease is present in plasma to cleave large multimers of von-Willebrand factor (vWF) into smaller less thrombogenic monomers especially in areas of high endothelial stress such as arterioles and capillaries. In familial form of TTP, there is a deficiency of ADAMTS-13 due to genetic mutations whereas in acquired form, there is a reduced activity of ADAMTS-13 due to the presence of plasma inhibitors (auto-antibodies). In the absence of adequate ADAMTS-13 activity, large vWF units attach to endothelium leading to the formation of microvascular thrombi rich in platelets. This thrombotic process is responsible for the thrombocytopenia, shear stress-induced hemolysis and organ dysfunction.

Acquired TTP usually occurs in a previously healthy individual although it can be seen in various other autoimmune diseases such as systemic lupus erythematosus (SLE). As evident in our case report, acquired TTP is seen more commonly in young adult females although other populations such as children and older adults can be affected as well.

Criteria for diagnosis of acquired TTP (Table 1) include ADAMTS-13 activity <10% and detectable anti-ADAMTS-13 antibodies in plasma [4]. This is because the complete 'pentad' associated with TTP was seen more commonly before the routine use of plasmapheresis. In addition, acute kidney injury is less common and milder in TTP as compared to other micro-angiopathies such as hemolytic-uremic syndrome (HUS). For these reasons, use of 'pentad' for diagnostic purposes has become obsolete. Our patient presented with thrombocytopenia, micro-angiopathic hemolytic anemia and acute kidney injury. HUS was initially considered as a probable diagnosis but TTP was confirmed based on ADAMTS-13 activity less than 5%. She still required temporary renal replacement therapy due to progressive kidney injury and oliguria.

**Table 1:** Diagnostic criteria for thrombotic thrombocytopenic purpura. TTP: Thrombotic thrombocytopenic purpura; MAHA: Microangiopathic hemolytic anemia; LDH: Lactate dehydrogenase; ADAMTS-13: A Disintegrin And Metalloprotease with ThromboSpondin type 1 motif-member 13.

CRITERIA	ACQUIRED TTP	FAMILIAL TTP
MAHA (Peripheral schistocytosis, elevated LDH, hyperbilirubinemia, negative indirect anti-globulin test)	Present	Present
Thrombocytopenia	Present	Present
Alternative causes of thrombocytopenia/hemolysis (drugs, infections, sepsis, malignancy)	Absent	Absent
ADAMTS-13 activity	<10%	<10%
Anti-ADAMTS-13 antibodies	Present	Absent
ADAMTS-13 gene mutations	Absent	Present

Presence of myocardial and liver injury in our patient indicates that a wide spectrum of organ dysfunction can be seen in TTP as demonstrated in Table 2 [5-7]. Autopsy findings in patients who died due to TTP revealed hyaline rich microvascular thrombi present in all organs [2]. Although it is a thrombotic condition, bleeding in the form of petechiae and purpura can also occur in TTP secondary to thrombocytopenia and vascular wall injury.

**Table 2:** Organ involvement seen in thrombotic thrombocytopenic purpura.

ORGANS INVOLVED	CLINICAL FEATURES
Central nervous system	Headache, altered mentation, coma, blindness, seizures, ischemic stroke, reversible posterior encephalopathy syndrome
Cardiovascular system	Acute coronary syndrome, arrhythmias, cardiomyopathy, cardiac arrest
Gastrointestinal system	Non-specific abdominal pain, mesenteric ischemia, ischemic hepatic injury, pancreatitis, colitis
Skin	Bruising, petechiae, purpura
Kidney	Elevated creatinine, abnormal urinalysis, rarely anuria
Pulmonary system	Acute respiratory distress syndrome

In contrast to microvascular involvement, large-vessel thrombosis is not a typical clinical manifestation of TTP. However, ischemic stroke has been reported to occur in the context of severe TTP [7]. Such patients are also prone to developing shock which can either be cardiogenic or distributive in nature [8,9]. This is similar to our patient who during the clinical course developed an acute ischemic infarct of basal ganglia and then cardiogenic shock which required temporary vasopressor therapy.

Management of an acute episode of TTP consists of plasmapheresis which replenishes ADAMTS-13 activity by removing inhibitor antibodies from the plasma. In addition, corticosteroids are given as adjuvant therapy to decrease the production of these antibodies [4]. However, 10% of patients with TTP will have an incomplete or no response to this management due to refractory disease. Refractory TTP is characterized by any of the following: development of new neurological abnormalities while on plasmapheresis, exacerbation of symptoms or laboratory findings while on plasmapheresis or failure to respond to plasmapheresis in form of improvement in platelet counts within the first four days. In such cases, Rituximab can be used which is a chimeric monoclonal antibody against CD-20. Rituximab has shown efficacy in refractory TTP when used in combination with plasmapheresis although this evidence comes from observational studies [10]. Nonetheless, the response to Rituximab has been found to be so effective that it is being suggested to be used as a first line agent along with plasmapheresis to decrease the incidence of refractoriness or relapses. Our case report is a perfect example of this recommendation when the patient deteriorated neurologically and hemodynamically despite being on plasmapheresis and corticosteroids. Rituximab infusions were thus initiated which led to a dramatic response and improvement within days.

## Conclusions

Our case report is interesting in many ways because it depicts various atypical manifestations of acquired TTP such as oliguric kidney injury requiring RRT (Renal replacement therapy), large-vessel thrombosis and cardiogenic shock. It also demonstrates that Rituximab is an efficacious agent in the management of severe and refractory cases of TTP.
